# Flanged Bombardier beetles from Shanghai, China, with description of a new species in the genus *Eustra* Schmidt-Goebel (Coleoptera, Carabidae, Paussinae)

**DOI:** 10.3897/zookeys.740.20458

**Published:** 2018-02-27

**Authors:** Xiao-Bin Song, Liang Tang, Zhong Peng

**Affiliations:** 1 Department of Biology, Shanghai Normal University, 100 Guilin Road, Xuhui District, Shanghai, 200234, P. R. China

**Keywords:** China, *Ectomomyrmes*, *Eustra*, *Itamus*, Paussinae, myrmecophilous, new species, *Pheidole*, *Platyrhopalus*, Shanghai

## Abstract

Four paussine species belonging to three different genera are discovered in Shanghai. A new species, *Eustra shanghaiensis* Song, **sp. n.**, is described, illustrated, and distinguished from the treated congeners. New distributional data or biological notes on *Eustra chinensis* Bänninger, 1949, *Itamus castaneus* Schmidt-Goebel, 1846, and *Platyrhopalus davidis* Fairmaire, 1886 are provided.

## Introduction

The ground beetle subfamily Paussinae Latreille, 1807 currently contains more than 30 species from China (Nagel 2003; Wrase and Schmidt 2007; Guéorguiev 2014; Maruyama 2014, 2016; Song et al. 2017; Wang 2017), among which only one is known to occur in Shanghai: *Eustra chinensis* Bänninger, 1949.

During several recent collecting trips conducted in Shanghai, the senior author and his colleagues collected a large series of paussine specimens. The examination of the specimens revealed a new species of ozaenine genus *Eustra* and three known species.

According to the latest revision of *Eustra* (Deuve, 2001), the genus contains two species from China: *E. chinensis* (Shanghai, Taiwan; Type locality: Shanghai, China), *E. taiwanica* Deuve, 2001 (Taiwan; Type locality: Taiwan, China). In 2014, Guéorguiev described the first Chinese troglobitic *Eustra* species, *E. petrovi* from Xianrendong, Yunnan. In this paper, a new *Eustra* species is described from Shanghai, illustrations provideded of all Shanghainese paussines, and biological information about the habitats and behaviors of *Eustra shanghaiensis* sp. n., *Eustra chinensis*, *Platyrhopalus davidis*, and *Itamus castaneus* observed in nature and captivity are provided.

## Materials and methods

Material used in this study is deposited in the following public and private collections:

**SNUC** Insect Collection of Shanghai Normal University, Shanghai, China;

**SNHM** Shanghai Natural History Museum;

**KUM** The Kyushu University Museum, Fukuoka;

**MNHN** The French National Museum of Natural History;

**cBWX** private collection of Wen-Xuan Bi, Shanghai, China;

**cLW** private collection of Wei Liu, Zhejiang, China;

**cJRX** private collection of Ri-Xing Jiang, Shandong, China;

**cSXB** private collection of Xiao-Bin Song, Shanghai, China;

**cWYX** private collection of Yong-Xiang Wu, Shanghai, China;

**cYZZ** private collection of Zhi-Zhou Yu, Shanghai, China.

The following abbreviations are applied in the text:

**BL** body length, from the anterior margin of the head to the apices of elytra;

**HW** head width, maximum width of the head;

**AL** length of antenna;

**ACL** maximum length of antennal club;

**ACW** maximum width of antennal club;

**PL** length of the pronotum along the midline;

**PW** maximum width of pronotum;

**EL** length of the elytra along the suture.

All measurements are in millimeters.

## Taxonomy

### Subfamily Paussinae Latreille, 1807

棒角甲亚科

#### Tribe Ozaenini Hope, 1838

折缘粗角步甲族

##### Subtribe Eustrina Jeannel, 1946

双斑粗角步甲亚族

###### Genus *Eustra* Schmidt-Gobel, 1846

双斑粗角步甲属

####### Eustra shanghaiensis

Taxon classificationAnimaliaColeopteraCarabidae

Song sp. n.

http://zoobank.org/26391AC8-3D78-41C6-BA55-F3322740E088

**Figure 1.**
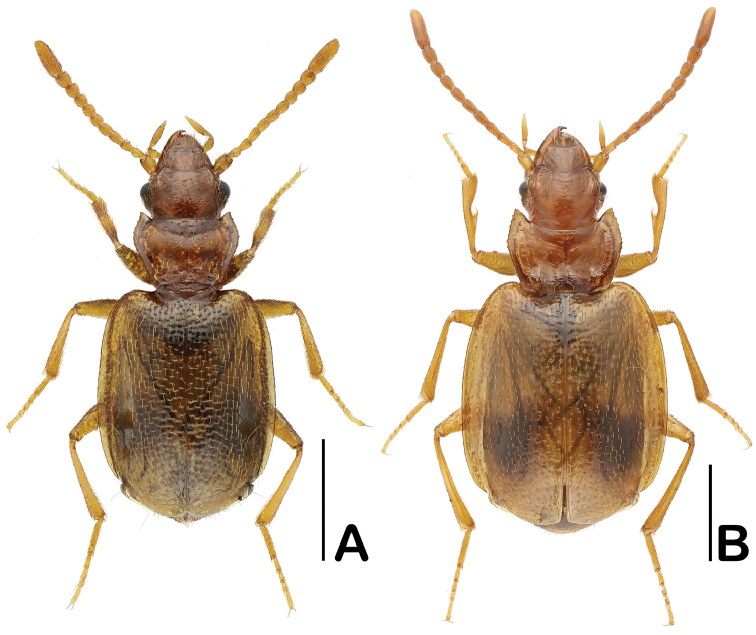
Dorsal habitus of *Eustra* species. **A** *E. shanghaiensis* sp. n., male (paratype) **B** *E. chinensis* Bänninger, 1949, female. Scale bars: 1 mm.

**Figure 2.**
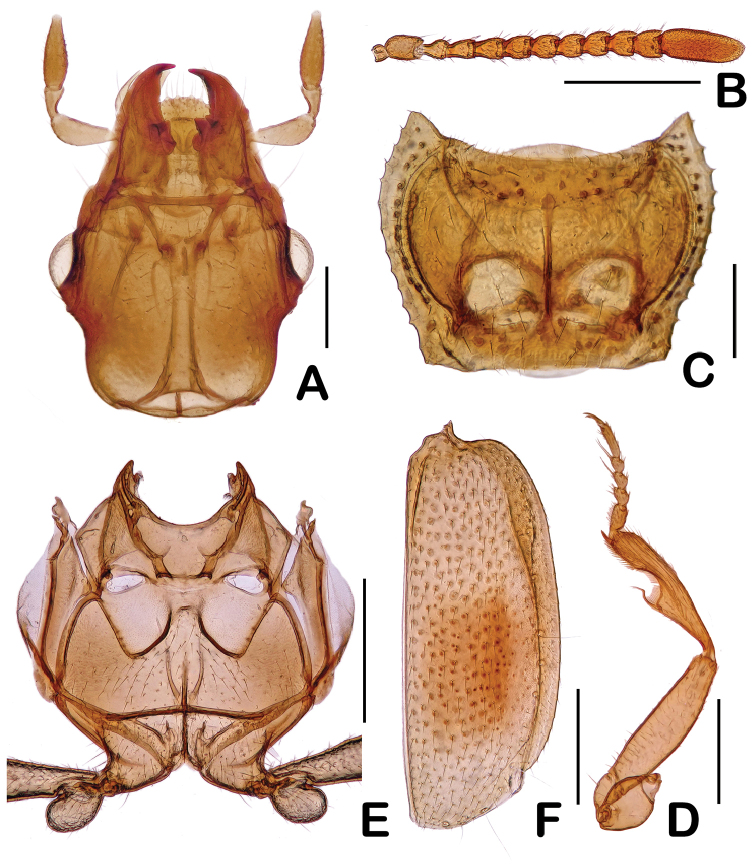
Diagnostic features of *Eustra shanghaiensis* sp. n. **A** Head **B** Antenna **C** Prothorax **D** Foreleg **E** Pterothorax **F** Elytron. Scale bars: 0.2 mm (**A, C**); 0.5 mm (**B, E, F**); 0.4 mm (**D**).

**Figure 3.**
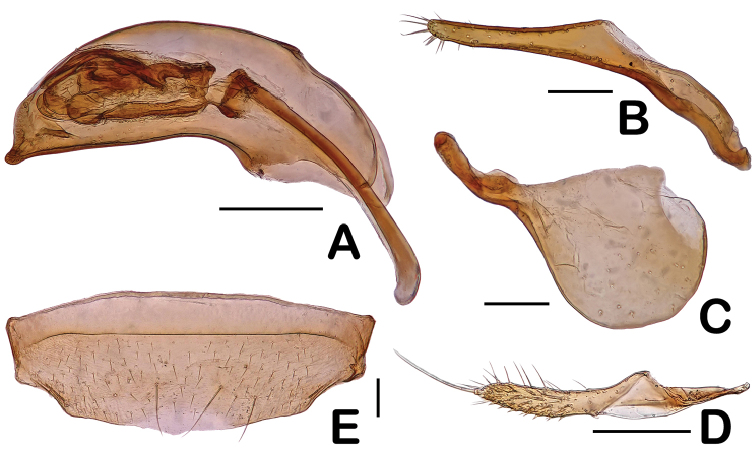
Diagnostic features of *Eustra shanghaiensis* sp. n. **A** Aedeagus, dorsal view **B** Right paramere **C** Left paramere **D** Gonopod IX **E** Male Sternite VII. Scale bars: 0.2 mm (**A–D, F**); 0.1 mm (**E**).

Figs 1A , 2 , 3 , 6A 上海双斑粗角步甲

######## Type material.

**Holotype**. ♂, (SNUC), labeled ‘CHINA: Shanghai, Pudong New District, Shanghai Binjiang Forest Park (上海滨江森林公园), 31°23'25"N, 121°22'10"E, alt. 5 m, 7.v.2017, Song, Peng, Hu, Wang & Liu leg. / HOLOTYPE [red], *Eustra shanghaiensis* sp. nov., Song det.2017’.

**Paratypes**. 3♂♂, 3♀♀, (SNHM), same data as holotype; 2♂♂, 2♀♀, (KUM), ditto; 1♂, 1♀, (MNHN), ditto; 5♂♂, 5♀♀, 84 exs, (SNUC), ditto; 74 exs, (cSXB), same data as holotype, but 27.vii.2017, Song, Zhou, Wang, Wang & Zhang leg; 11 exs, (cSXB), same data as holotype, but 24.ix.2016, Zhong Peng leg; 1 ex (cSXB), labeled ‘CHINA: Shanghai, Changning District, Tianshan Park (天山公园), 31°12'45"N, 121°24'10"E, alt. 14 m, 20.iv.2008, Xiao-Bin Song leg.; 1ex, (cSXB), ditto, but 12.iv.2008; 1 ex, (cSXB), ditto, but iv.2008; 1 ex, (cBWX), labeled ‘CHINA: Shanghai, Pudong New District, nr. Zhangjianggaoke (张江高科), 31°11'84"N, 121°34'82"E, alt. 4 m, 28.iii.2006, Wen-Xuan Bi leg.; 8 exs, (cSXB), ditto, but 5.ii.2009, Song & Ding leg. / all paratypes, labeled ‘Paratype [yellow], *Eustra shanghaiensis* sp. nov. Song det. 2017’.

######## Comparative notes.

*Eustra shanghaiensis* sp. n. is closely allied to *E. hammondi* Deuve, 2001 from Mindanao, Philippines in sharing similar body size, general habitus and aedeagal structure (Figs 1A, 3A). The new species can be readily separated from latter by the pronotal front angles strongly produced, the wider aedeagus, the apex of aedeagal median lobe much shorter and wider and the relatively long apical portion of right paramere. It differs from its Shanghainese congener *E. chinensis* by the smaller body size and the different shape of aedeagus.

######## Description.

Body (Fig. 1A) 3.06–3.17 mm; yellowish-brown, head and pronotum somewhat reddish; each elytron with a dark spot.

Head (Fig. 2A) convex, gently covered with yellow setae, microsculpture faint; fully carinate near eyes; clypeus anteriorly gently concave, with 2 pairs of long setae at anterior margin; labrum with anterior margin minutely denticulate, with 12–14 long setae; Eyes somewhat small; antennae (Fig. 2B) submoniliform, with antennomeres I and II clavate, increasing in diameter distally gradually; antennomere I somewhat shorter than 2nd and 3rd combined; antennomeres V–X almost as wide as long; antennomere XI evidently longer than the 1st.

Pronotum (Fig. 2C) sparsely covered with yellow setae; distinctly wider than long, widest at apical third; moderately contracted anteriorly and posteriorly; disc moderately convex medially and reflexed on lateral sides; front angles strongly produced; midline distinct, almost reaching both anterior and posterior borders.

Pterothorax shaped as in Fig. 2E, meso-coxae disjunct, meta-coxae separated in midline of body.

Elytra (Fig. 2F) densely punctulate and pubescent, distinctly wider than prothorax; shoulders rounded and not bordered; each side with an obscurely dark spot; surface moderately covered with short setae, but along the right side of dark spot glabrous.

Hind wings well developed.

Legs (Fig. 1A) relatively long and slender; both spurs of pro-tibiae (Fig. 2D) are terminal, almost equal in length.

Male. Sternite VII (Fig. 3E) wide, widely truncate, slightly acute at middle, with 4 long setae near apex. Median lobe of aedeagus shaped as in Fig. 3A, ends in a blunt tip, with a spoon-shaped sclerite on endophallus; right paramere (Fig. 3B) arcuate, apical portion narrow and elongate; left paramere shaped as in Fig. 3C, large, almost glabrous, rounded at apex.

Female. Gonopod IX shaped as in Fig. 3D.

######## Comments.

Moore et al. 2011 described and illustrated the larval structure of *Eustra chinensis* based on larval specimens collected together with some adults from Tianshan Park, Shanghai with no association ants (Fig. 6A). However, all these specimens are now reconsidered as larvae of the new species, *Eustra shanghaiensis* sp. n. described here.

######## Measurements.

BL, 3.06–3.17; HW, 0.71–0.76; PL, 0.52–0.57; EL, 1.89–1.92.

######## Distribution.

China: Shanghai.

######## Biological notes.

Both adults and larvae are collected under rotten wood or bark during the whole year in Shanghai.

######## Symbiotic host.

Free living, not associated with ant.

######## Etymology.

Named after its type locality of Shanghai Latinized.

####### Eustra chinensis

Taxon classificationAnimaliaColeopteraCarabidae

Bänninger, 1949

Figs 1B , 6B 中华双斑粗角步甲

Eustra chinensis Bänninger, 1949: 134 (original description, type locality: Shanghai, China); Deuve 2001: 570 (diagnosis, new record from Taiwan, China); Teradaet al. 2013: 31 (redescription); Maruyama et al. 2013: 2 (associated with Ectomomyrmes javana).

######## Material examined.

1♂, 1♀, (cSXB), labeled ‘CHINA: Shanghai, Xuhui District, Shanghai Normal University (上海师范大学), 31°09'48"N, 121°24'45"E , alt. 4 m, 11.V.2017, Xiao-Bin Song leg., [from colony of *Ectomomyrmes javana*]’; 1♂, 1 ex, (cSXB), ditto, but 20.ix.2016, Zhong Peng leg.; 1 ex, (cJRX), ditto; 1 ex, (cSXB), labeled ‘CHINA: Shanghai, Changning District, Zhongshan Park (中山公园), 31°13'25"N, 121°25'00"E, alt. 9 m, xi.2006, Xiao-Bin Song leg.’

######## Other material examined.

**Zhejiang**: 1ex, (cSXB), labeled ‘CHINA: Zhejiang, Hangzhou City, Lin’an District, West Tianmushan (西天目山), 30°19'28"N, 119°26'54"E , alt. 380 m, 16.vii–9.viii.2017, Xiao-Bin Song leg., [F. I. T.].’

######## Comments.

*Eustra chinensis* is characterized by the large body size and the broad elytra. This is the only known myrmecophilous species of the genus *Eustra*. Adults are collected from *Ectomomyrmes javana* (Mayr, 1867) nests under the stone (Fig. 6B), and have been observed feeding on dead insects inside nest of *E. javana* (Maruyama et al., 2013). Wendy et al. (2011) described an unidentified *Eustra* larva which collected during the excavation of nest of *Ectomomyrmes javana* in Taiwan, based on its same special symbiotic host and the reasonable distribution, the larva should belongs to *Eustra chinensis*.

######## Measurements.

BL, 4.03–4.30; HW, 0.94–0.96; PL, 0.65–0.68; PW, 1.21–1.25; EL, 2.32–2.61.

######## Distribution.

China: Shanghai, Zhejiang (new provincial record), Taiwan; Japan: Yaeyama-shoto.

######## Symbiotic host.

*Ectomomyrmes javana* (Mayr, 1867) (Figs 6B, 7A, B).

##### Subtribe Ozaenina Hope, 1838

折缘粗角步甲亚族

###### Genus *Itamus* Loew, 1849

伊塔粗角步甲

####### Itamus castaneus

Taxon classificationAnimaliaColeopteraCarabidae

Schmidt-Goebel, 1846

Figs 4 , 6C 栗伊塔粗角步甲

Itamus castaneus Schmidt-Goebel, 1846: 67 (original description, type locality: Myanmar); Zhao and Tian 2003: 57 (new record from Guangdong, China).

######## Material examined.

1♂, (cSXB), labeled ‘CHINA: Shanghai, Changning District, nr. Zhongshan Park (中山公园), 31°12'59"N, 121°25'17"E, alt. 11m, 28.vii.2017, Xiao-Bin Song leg.’; 3♀♀, (cSXB), ditto, but 22.viii.2017; 4 exs, (cSXB), ditto, but 25.viii.2017; 1 ex, (cSXB), ditto, but 7.ix.2017; 2 exs, (cSXB), ditto, but 27.ix.2017; 1 ex, (cWYX), labeled ‘CHINA: Shanghai, Jing’an District, nr. Jiang’an Temple (静安寺), 10.viii.2017, Yong-Xiang Wu leg.’; 1 ex, (cWYX), ditto, but vii.2017.

**Figure 4.**
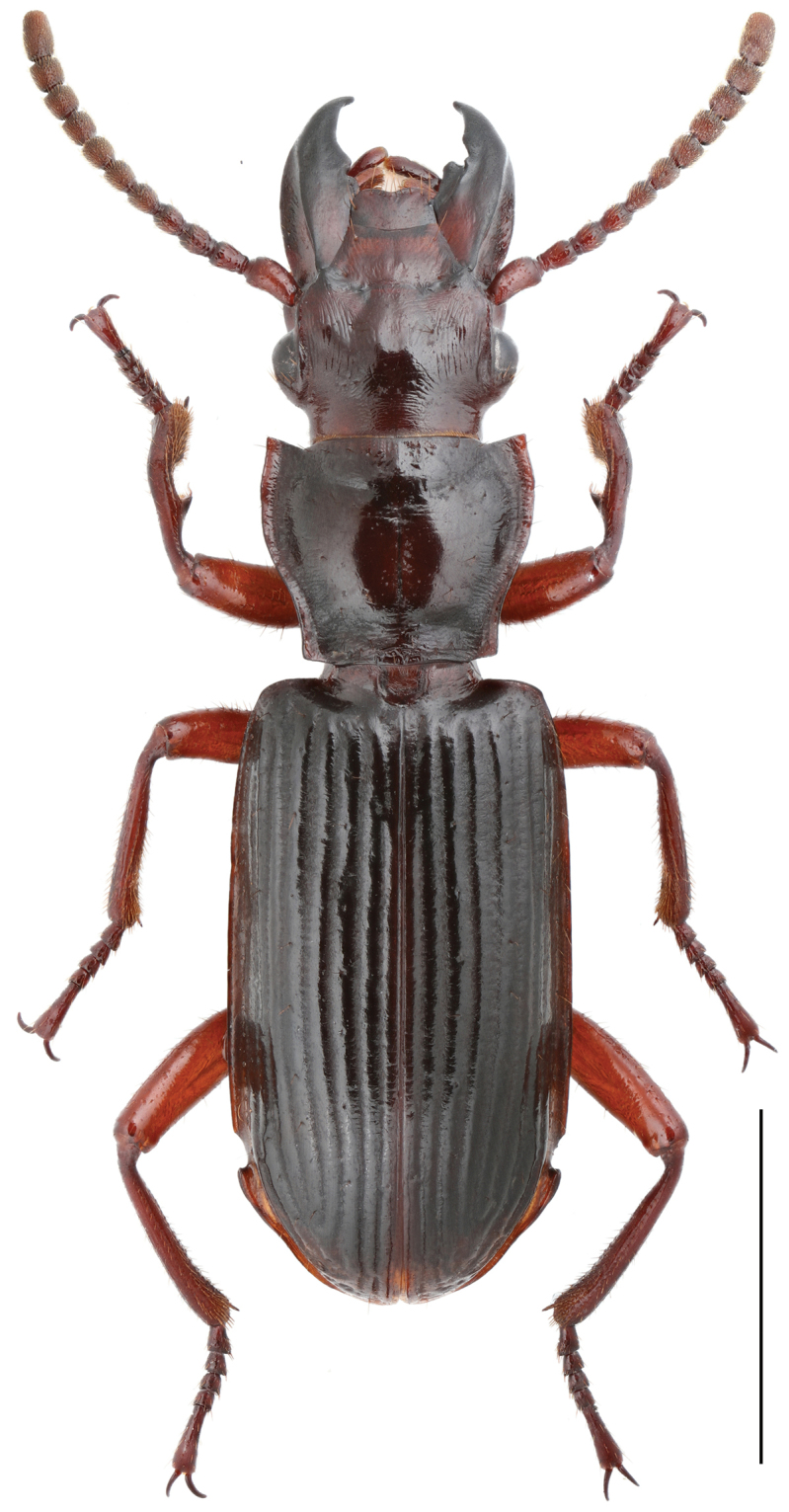
*Itamus castaneus* Schmidt-Goebel, 1846, Male. Scale bar 5 mm.

######## Other material examined.

Yunnan: 1ex, (cSXB), labeled ‘CHINA: Yunnan, Xishuangbanna, Xishuangbanna Tropical Botanical Garden (西双版纳植物园), iv-2009, Xiao-Yu Zhu leg.’; Fujian: 1 ex, (cSXB), labeled ‘CHINA: Fujian, Nanping City, Wuyishan (武夷山), Yunvfeng (玉女峰), Zu-Qi Mai leg., under rotten wood.’; Zhejiang: 1 ex, (cLW), labeled ‘CHINA: Zhejiang, Hangzhou, Hangzhou Botanical Gardern, Taoyuanling (桃源岭), viii-2017, Wei Liu leg.’; 3 exs, (cLW), ditto, but Pujiaxincun (濮家新村), vi-2015, on *Broussonetia papyrifera* trees.

######## Comments.

Zhao and Tian (2003) first recorded the ozaenine genus *Itamus* Loew, 1849 in China, with two known Asian species, i.e., *I. castaneus* from Guangdong and *I. dentatus* Andrews, 1919 from Guangxi. *Itamus castaneus* can be readily separated from its Chinese congener *I. dentatus* by the larger body size, shoulders of elytra almost without denticle and the fore-femur with an obvious projection. All specimens from Shanghai were founded walking on the ground or at light in July to October (Fig. 6C), individuals are observed to feed on amber snails (*Suecinea* sp. Draparnaud) and dead cave cricket (Rhaphidophoridae). One specimen from Fujian was collected under decaying wood (Mai per. comm.). Three individuals from Zhejiang are collected at night on the trunks of *Broussonetia papyrifera* (L.) L’Hér. ex Vent. trees (Liu per. comm.).

######## Measurements.

BL, 15.62; HW, 3.61; PL, 3.22; PW, 4.28; EL, 8.90.

######## Distribution.

China: Shanghai (new provincial record), Zhejiang (new provincial record), Fujian (new provincial record), Guangdong, Yunnan (new provincial record); Myanmar; Laos; Sri Lanka; Thailand.

######## Symbiotic host.

Free living, not associated with ant.

#### Tribe Paussini Latreille, 1807

棒角甲族

##### Subtribe Paussina Latreille, 1807

棒角甲亚族

###### Genus *Platyrhopalus* Westwood, 1833

圆角棒角甲属

####### Platyrhopalus davidis

Taxon classificationAnimaliaColeopteraCarabidae

Fairmaire, 1886

**Figure 5.**
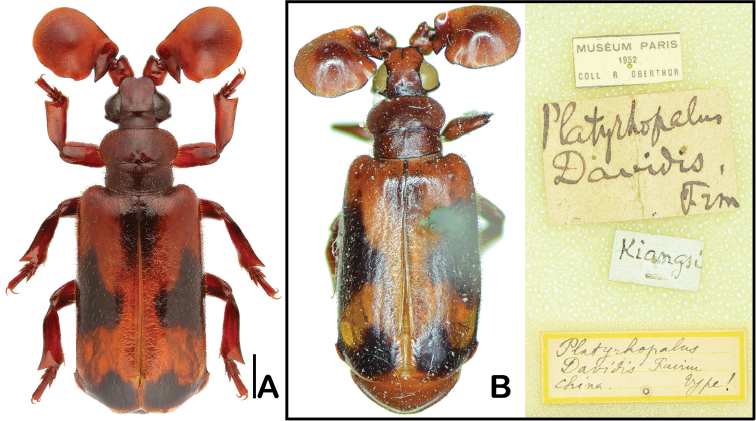
*Platyrhopalus davidis* Fairmaire, 1886. **A** Individual from Shanghai Botanical Gardern **B** Holotype. Scale bar 1 mm.

**Figure 6.**
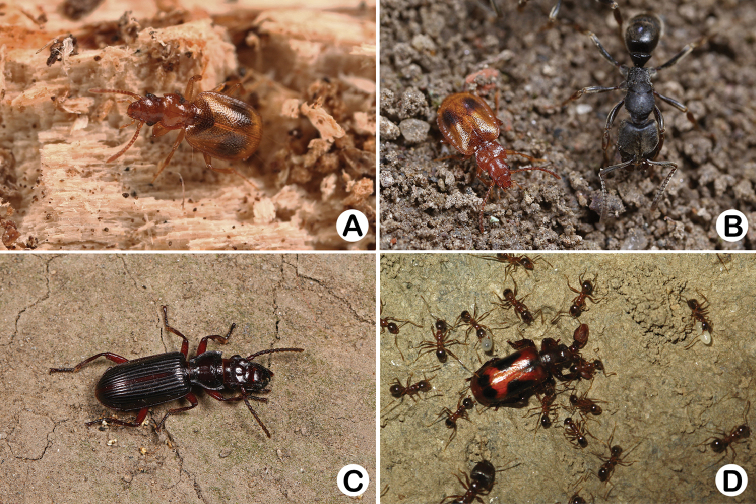
Habitus of Shanghainese paussines. **A** *Eustra shanghaiensis* sp. n. found in rotten wood **B** *Eustra chinensis*, with a work of *Ectomomyrmes javana* **C** *Itamus castaneus*, walking on the ground at night **D** *Platyrhopalus davidis*, associated with *Pheidole* ants. Photographs by Xiao-Bin Song (**A–C**) and Wen-Xuan Bi (**D**).

Figs 5A , 6D 大卫圆角棒角甲

Platyrhopalus davidis Fairmaire, 1886: 224 (original description, type locality: Kiang-si = Jiangxi, China); Luna de Carvalho 1987: 390 (diagnosis).

######## Material examined.

1♂, 1ex, (cSXB), labeled ‘上海植物园, 3-XI-2007, 毕文烜’; 1ex, ditto, but, 21-IV-2007, pinned with *Pheidole* ant (1 soldier, 3 workers); 1♀, (cSXB), labeled ‘SH. Botanical Gardern Xuhui District, Shanghai City, 27-VII-2007’; 1 ex, (cSXB), ditto, but 25-VI-2008; 1 ex, (SNUC), labeled ‘CHINA: Shanghai, Fengxian District, Shanghai Normal Univeristy (上海师范大学), 30°50'09"N, 121°31'09"E, alt. 6 m, 15.vi.2007, Xiao-Yu Zhu leg.’; 1 ex, (SNUC), ditto, but 20.v.2008, Yu-Di Wang leg.; 1 ex, (SNUC), ditto, but 1.2008; 1♀, (cSXB), labeled ‘ CHINA: Shanghai, Fengxian District, Shanghai Institute of Technology (上海应用技术大学), 30°50'15"N, 121°30'20"E, alt. 5 m, vii.2011, De-Yao Zhou leg.’; 2 ex, (SNUC), labeled ‘CHINA: Shanghai, Chongming District, Dongtan N. R. (东滩), 7.v.2007, Jia-Yao Hu leg.’; 1♂, (SNUC), ditto, but 30.vi.2007; 1 ex, (SNUC), labeled ‘CHINA: Shanghai, Chongming District, Beihu (北湖), 1.vii.2008, Jia-Yao Hu leg.’, pinned with 6 *Tetramorium caespitum* workers; 1 ex (cYZZ), ditto, but Xitan (西滩), 15.vii.2007, Hong-Qiong Li leg.

######## Other material examined.

Anhui: 1ex, (cSXB), labeled ‘CHINA: Anhui, Fuyang City, Yingzhou District (颍州), Qiyuhedong Vill. (七渔河村), near dam, from ant nest, nr. 32°54'31"N, 115°46'29"E, 29-VI-2013, J-B Dong leg.’; Fujian: 1 ex, (SNUC), labeled ‘Mt. Wuyi, Fujian, Li-Zhen Li leg., 10~14-VII-2002’; Shandong: 1 ex, (cSXB), labeled ‘鲁, 莱阳, 旌旗山, 14.5.15., JRX.’; Hubei: 1 ex, (cSXB), labeled ‘湖北, 大店林场, 26.v.2016’ ; Hunan: 1 ex, (cSXB), labeled ‘湖南, 长沙, 1980.9, 灯下, 徐慧?’; Yunnan: 1 ex, (cSXB), labeled ‘CHINA: Hunan Province, Leiyang City (耒阳), vi-2011, Hao Xu leg.’; 1 ex, (cSXB), labeled ‘云南, 昭通, 黄华, 石水井—花椒地, 2007-8-13’; 1 ex, (cSXB), labeled ‘CHINA, Yunnan Prov., Yingjiang County (盈江县), Tongbiguan (铜壁关), alt. 1330 m, 23°36'N, 97°36'E , 23-V-2013, Chao Wu leg.’; Xizang: 1 ex, (cSXB), labeled ‘CHINA, Xizang, Linzhi, Motuo County, Beibeng Vill. (背崩乡), alt. 780 m, 10-viii-2010, Wen-Xuan Bi leg.’; 1 ex, (cSXB), labeled ‘CHINA, Xizang, Linzhi, Motuo County, Beibeng Vill. (背崩乡), 29.243469,95. 169677,769.01, 26-vi-2017, Jing-Song Shi leg.’.

######## Comments.

*Platyrhopalus davidis* is widely distributed in China, and specimens are often collected by light trap. Populations from Shanghai, Shandong, Hubei are recorded to be associated with *Pheidole* ants (Fig. 6D), but one individual from Beihu (北湖), Shanghai was founded with *Tetramorium* ants (Hu pers. comm.).

######## Measurements.

BL, 6.84–7.55; HW, 1.41–1.56; PL, 1.37–1.46; PW, 1.59–1.80; EL, 4.45–5.00; ACL, 1.70–1.87; ACW, 1.50–1.57.

######## Distribution.

China: Beijing, Shanxi, Shanghai (new provincial record), Jiangsu, Zhejiang (new provincial record), Anhui (new provincial record), Fujian, Jiangxi, Shandong (new provincial record), Henan, Hubei (new provincial record), Hunan, Guangdong (new provincial record), Sichuan, Guizhou, Yunnan (new provincial record), Xizang?, Shaanxi.

######## Symbiotic host.

**Figure 7.**
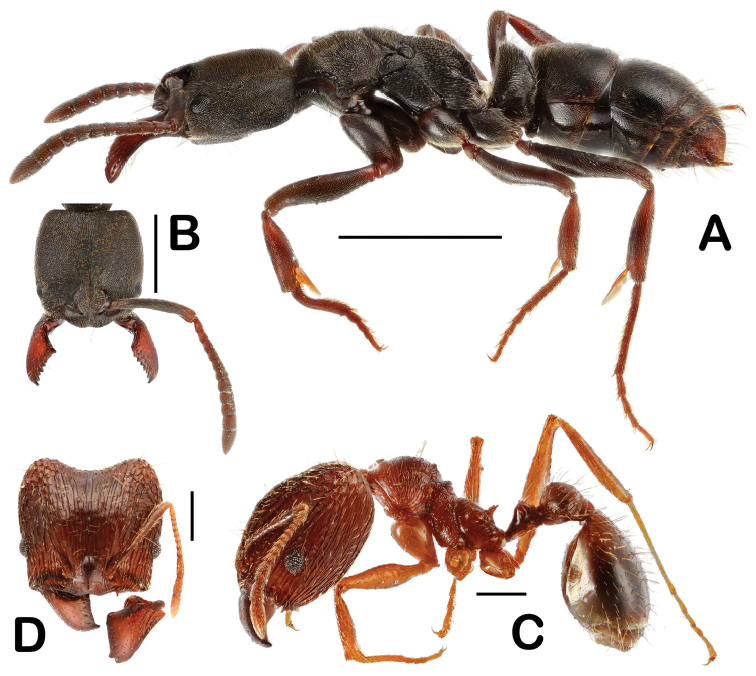
Host ants of *Eustra chinensis* and *Platyrhopalus davidis*. **A** *Ectomomyrmes javana* (Mayr, 1867), body, lateral view **B** ditto, but head, dorsal view **C** *Pheidole* sp., soldier, lateral view **D** ditto, but head, dorsal view. Scale bars: 2 mm (**A**); 1 mm (**B**); 0.5 mm (**C, D**).

*Pheidole* sp. (Figs 6D, 7C, D).

## Supplementary Material

XML Treatment for Eustra shanghaiensis

XML Treatment for Eustra chinensis

XML Treatment for Itamus castaneus

XML Treatment for Platyrhopalus davidis
